# Supplementary material for: SIRT7 is a histone desuccinylase that functionally links to chromatin compaction and genome stability
Source: Nat Commun. 2016 Jul 20;7:12235. doi: 10.1038/ncomms12235 (PMC4961794; doi:10.1038/ncomms12235)
Supplement: Supplementary Information — Supplementary Figures 1-11 and Supplementary Tables 1-4 [file ncomms12235-s1.pdf]

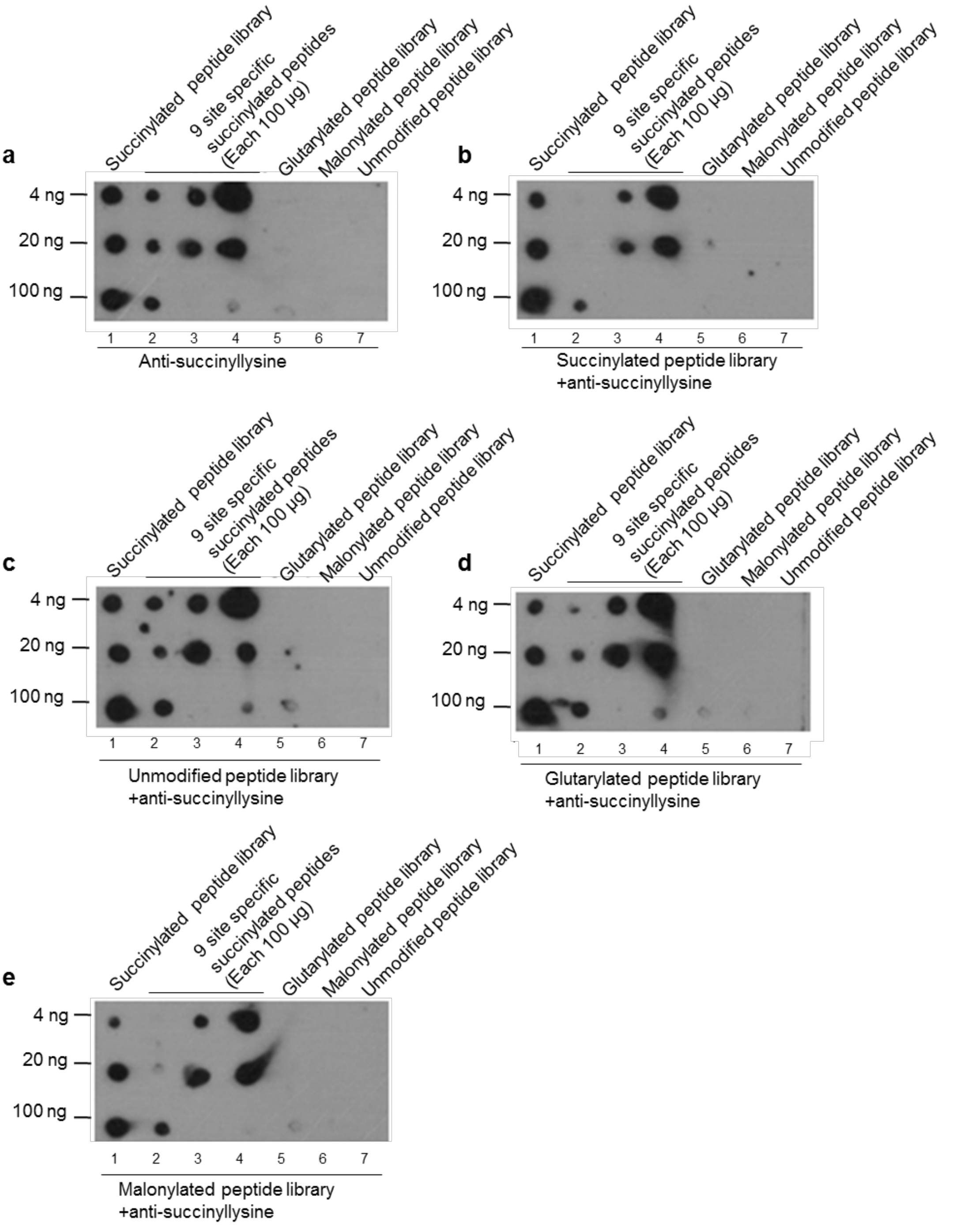


**Supplementary Figure 1. Verification of the specificity of anti-succinyllysine rabbit antibodies by dot blotting.**

(**a-e**) The serial diluted succinylated peptides library (Lane 1), glutarylated peptide library (Lane 5), malonylated peptide library (Lane 6), unmodified peptide library (Lane 7) and 9 site specific succinylated peptides (Lane 2- Lane 4, each 100 ng ) were dotted on the PVDF membrane as indicated, and then probed with the

anti-succinyllysine rabbit antibody alone (**a**), anti-succinyllysine rabbit antibody pre-adsorbed with 50 ng of succinylated peptide library (**b**), anti-succinyllysine rabbit antibody pre-adsorbed with 50 ng of unmodified peptide library (**c**), anti-succinyllysine rabbit antibody pre-adsorbed with 50 ng of glutarylated peptide library (**d**), or anti-succinyllysine rabbit antibody pre-adsorbed with 50 ng of malonylated peptide library (**e**).

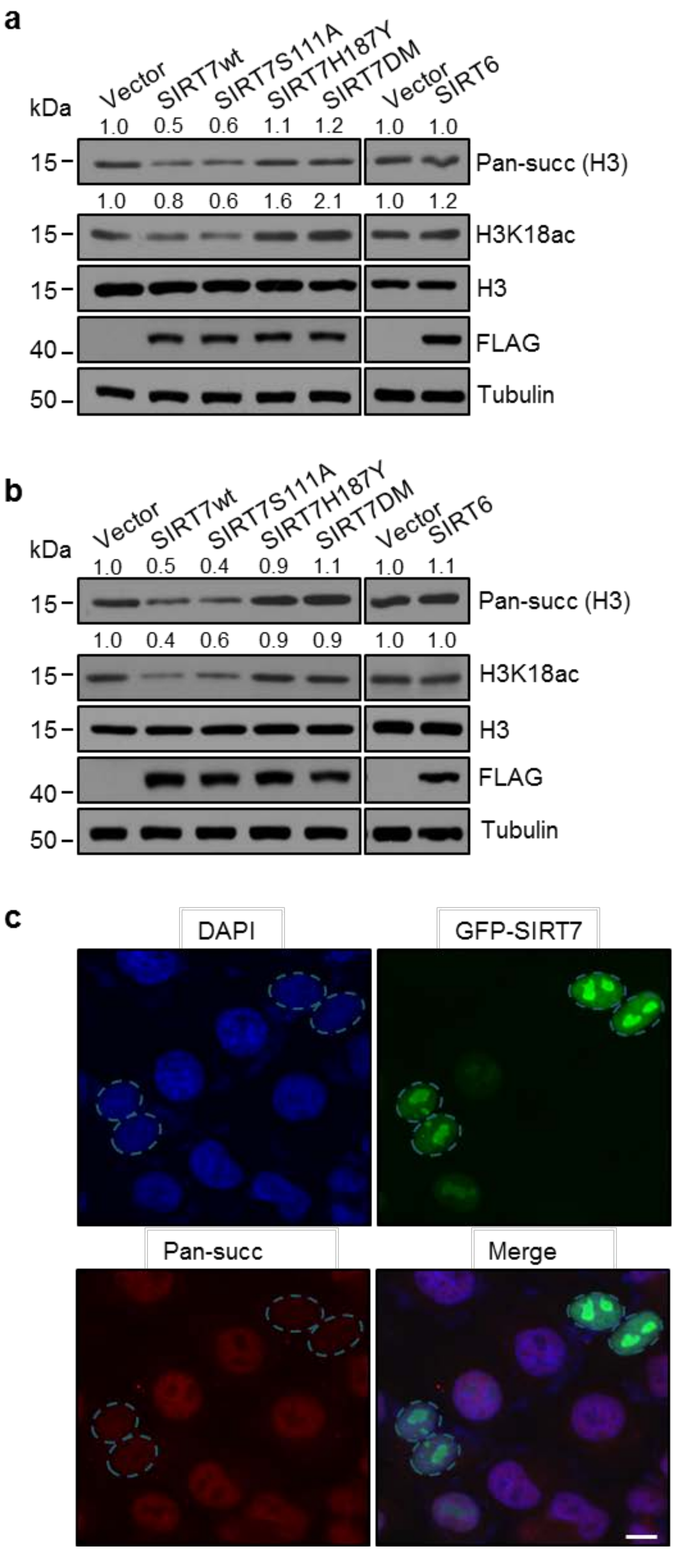


**Supplementary Figure 2. SIRT7 regulates histone succinylation.**

(**a-b**) HEK293T cells were transfected with wild-type SIRT7, SIRT7 mutants, or SIRT6. Histones were extracted and pan-succinylation of H3 and H3K18ac were analyzed by western blotting. Whole cell lysate were prepared for monitoring the

efficiency of overexpression of SIRT7 and SIRT6 by western blotting. The bands were quantified with Image J software. The numbers above indicate the relative levels of the indicated modifications. (**c**) Immunofluorescence analysis of pan-succinylation level in control or GFP-SIRT7-overexpressed U2OS cells. Bar, 10 μm.

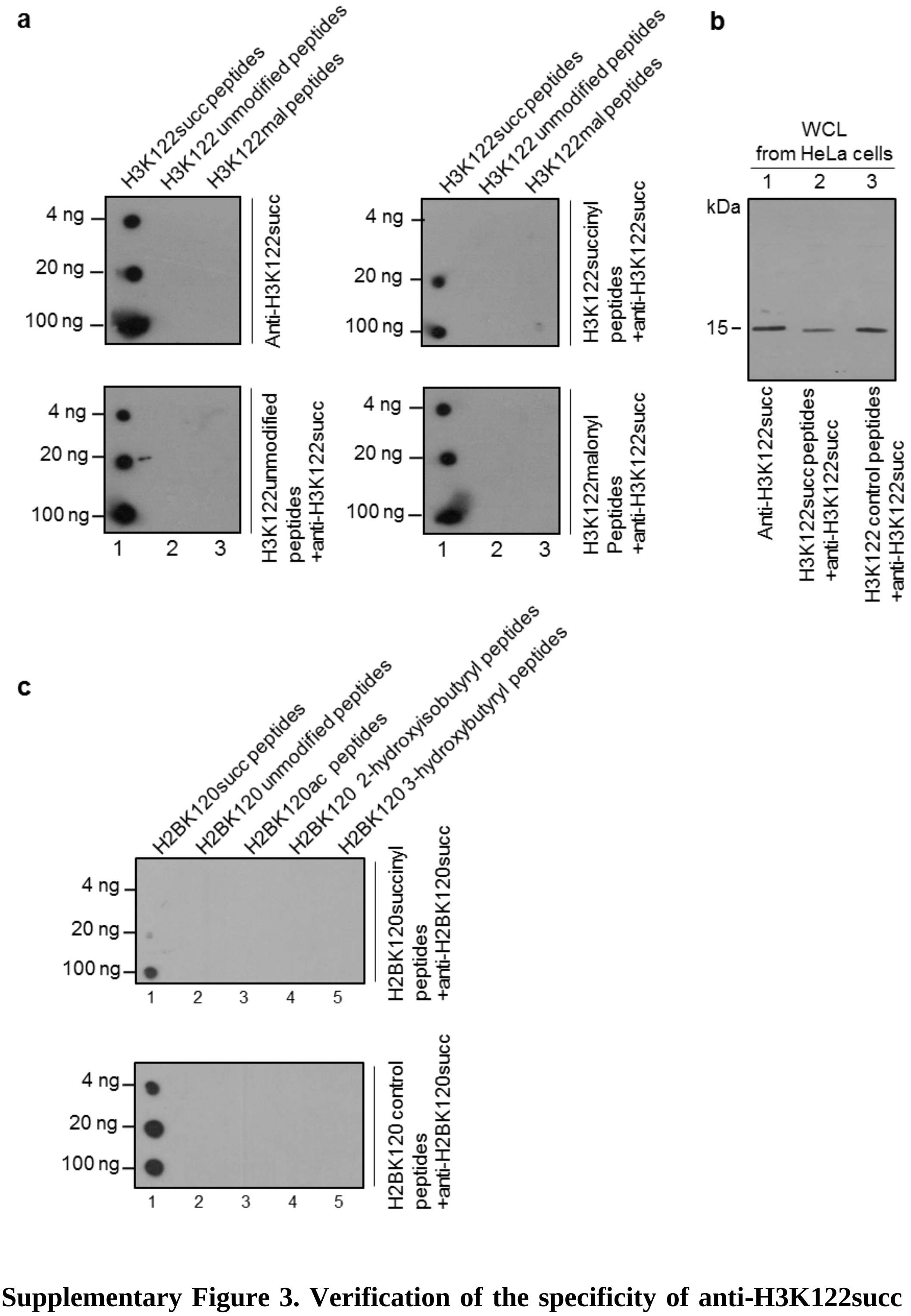


**Supplementary Figure 3. Verification of the specificity of anti-H3K122succ and anti-H2BK120succ antibodies.**

(**a**) The H3K122succinyl peptides (Lane 1), H3K122 unmodified control peptides (Lane 2) and the H3K122malonyl peptides (lane 3) were serial dotted on the PVDF membrane as indicated, and then probed with the anti-H3K122succ rabbit antibody alone, anti-H3K122succ rabbit antibody pre-adsorbed with 20 ng of H3K122succinyl

peptides, anti-H3K122succ rabbit antibody pre-adsorbed with 20 ng of H3K122 control peptides, or anti-H3K122succ rabbit antibody pre-adsorbed with 20 ng of H3K122malonyl peptides. (**b**) Whole cellular lysate from HeLa cells treated with sodium succinate were resolved on SDS-PAGE and were probed with the anti-H3K122succ rabbit antibody alone (Lane 1) or anti-H3K122succ rabbit antibody pre-adsorbed with 50 ng of H3K122succinyl peptides (Lane 2) or H3K122 control peptides (Lane 3). (**c**) The H2BK120succinyl peptides (Lane 1), H2BK120 unmodified control peptides (Lane 2), H2BK120acetyl peptides (lane 3), H2BK120 2-hydroxyisobutyryl peptides (lane 4) and H2BK120 3-hydroxybutyryl peptides (lane 5) were serial dotted on the PVDF membrane as indicated, and then probed with anti-H2BK120succ rabbit antibody pre-adsorbed with 100 ng of H2BK120succinyl peptides (upper), or H2BK120 control peptides (lower).

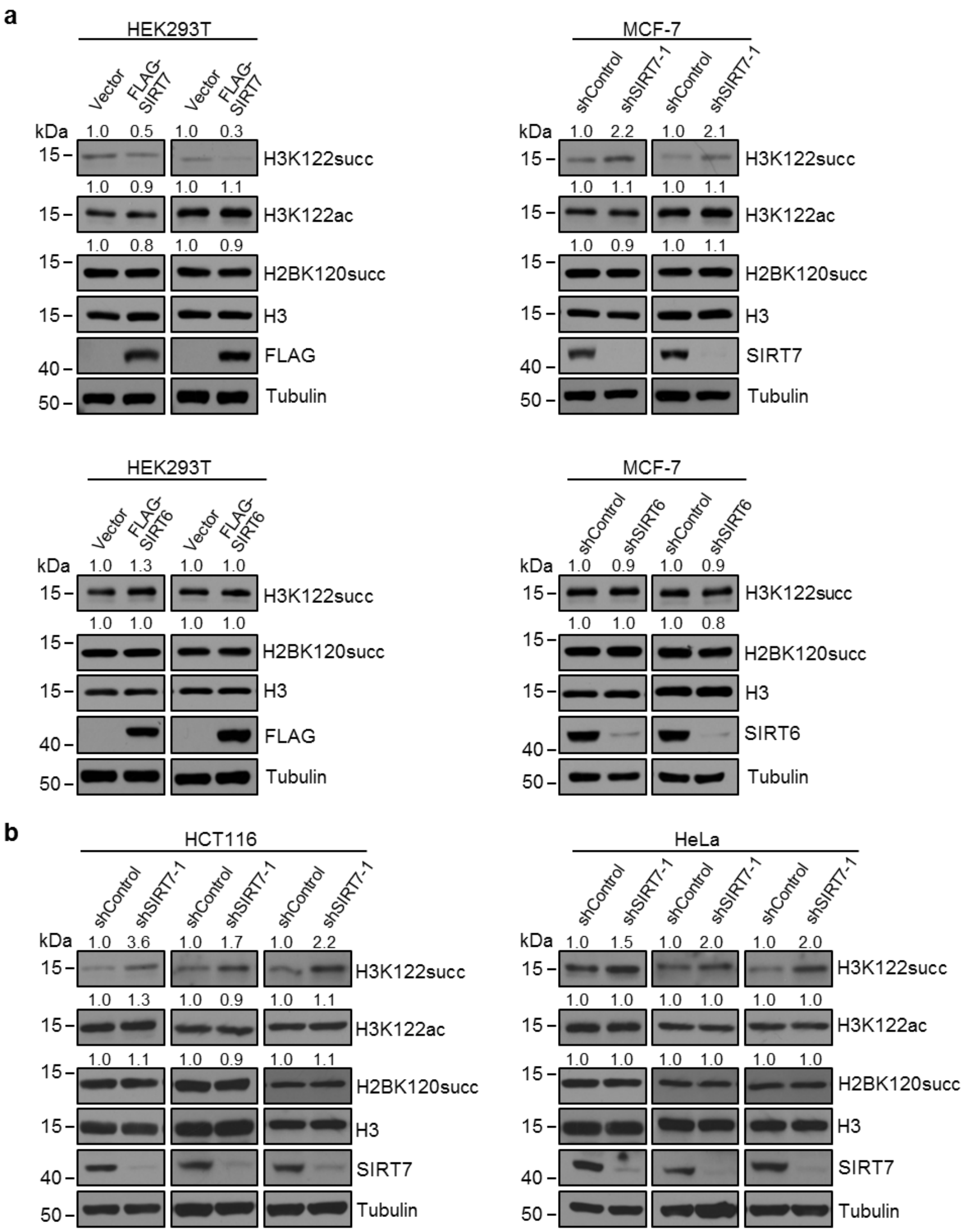

a
HEK293T
Vector
FLAG-SIRT7
kDa
1.0 0.5 1.0 0.3
H3K122succ
1.0 0.9 1.0 1.1
H3K122ac
1.0 0.8 1.0 0.9
H2BK120succ
H3
FLAG
Tubulin
15
40
50
MCF-7
shControl
shSIRT7-1
1.0 2.2 1.0 2.1
H3K122succ
1.0 1.1 1.0 1.1
H3K122ac
1.0 0.9 1.0 1.1
H2BK120succ
H3
SIRT7
Tubulin
HEK293T
Vector
FLAG-SIRT6
1.0 1.3 1.0 1.0
H3K122succ
1.0 1.0 1.0 1.0
H2BK120succ
H3
FLAG
Tubulin
MCF-7
shControl
shSIRT6
1.0 0.9 1.0 0.9
H3K122succ
1.0 1.0 1.0 0.8
H2BK120succ
H3
SIRT6
Tubulin
b
HCT116
shControl
shSIRT7-1
1.0 3.6 1.0 1.7 1.0 2.2
H3K122succ
1.0 1.3 1.0 0.9 1.0 1.1
H3K122ac
1.0 1.1 1.0 0.9 1.0 1.1
H2BK120succ
H3
SIRT7
Tubulin
HeLa
shControl
shSIRT7-1
1.0 1.5 1.0 2.0 1.0 2.0
H3K122succ
1.0 1.0 1.0 1.0 1.0 1.0
H3K122ac
1.0 1.0 1.0 1.0 1.0 1.0
H2BK120succ
H3
SIRT7
Tubulin

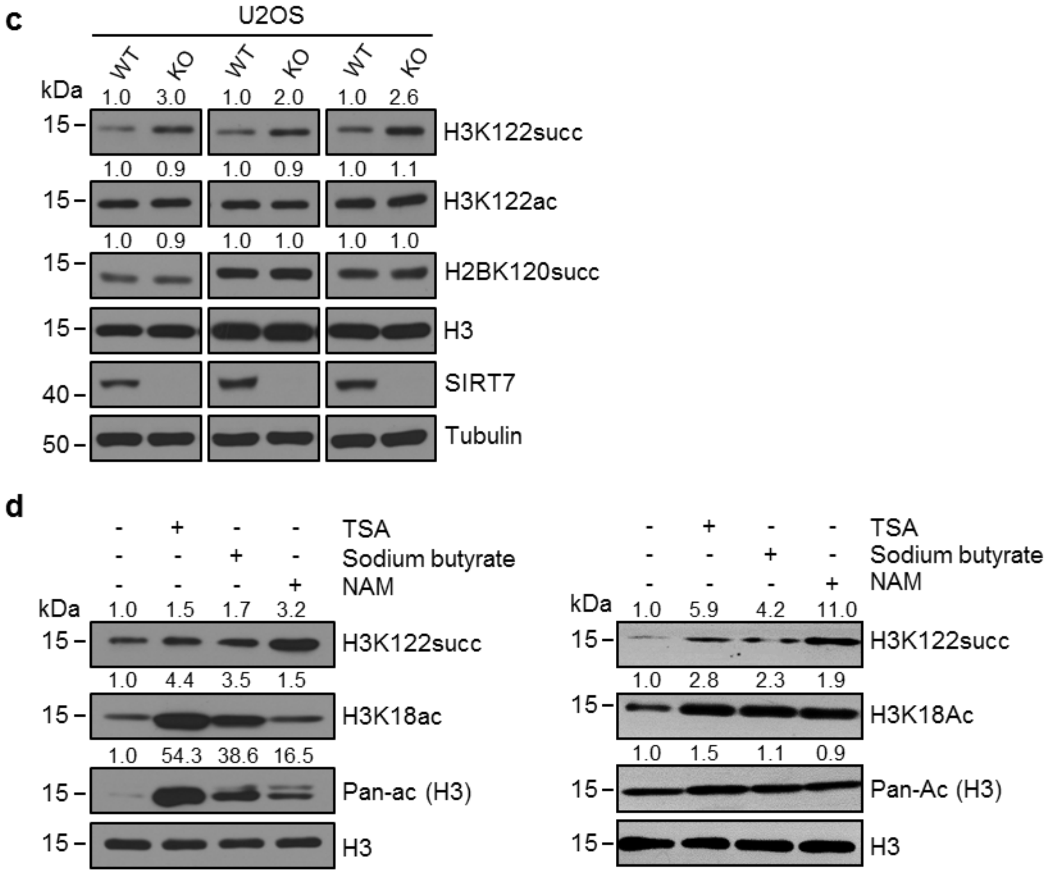


**Supplementary Figure 4. SIRT7 regulates H3K122succ *in vivo*.**

(**a**) SIRT7 or SIRT6 was overexpressed in HEK293T cells or knocked down in MCF-7 cells. Histones were extracted and succinylation and acetylation were analyzed by western blotting with the indicated antibodies. The efficiency of overexpression or knockdown of SIRT7 and SIRT6 was monitored by western blotting of whole cell lysate with corresponding ant ibodies. (**b**) SIRT7 was knocked down in HCT116 (left) or HeLa (right) cells. Histones were extracted and succinyl and acetyl modifications were analyzed by western blotting with the indicated antibodies. The efficiency of SIRT7 knockdown was monitored by western blotting of whole cell lysate with corresponding antibodies. (**c**) Histones from wild-type or SIRT7 knockout U2OS cells were extracted and analyzed by western blotting with the indicated antibodies. The efficiency of SIRT7 knockout was monitored by western blotting of whole cell lysate with corresponding antibodies. (**d**) MCF-7 cells were treated with 10 μM trichostatin A (TSA), 4 mM of sodium butyrate, or 10 mM nicotinamide (NAM). Twenty four hours later, cells were harvested and soluble histones were prepared and subjected to western blotting with antibodies as indicated.

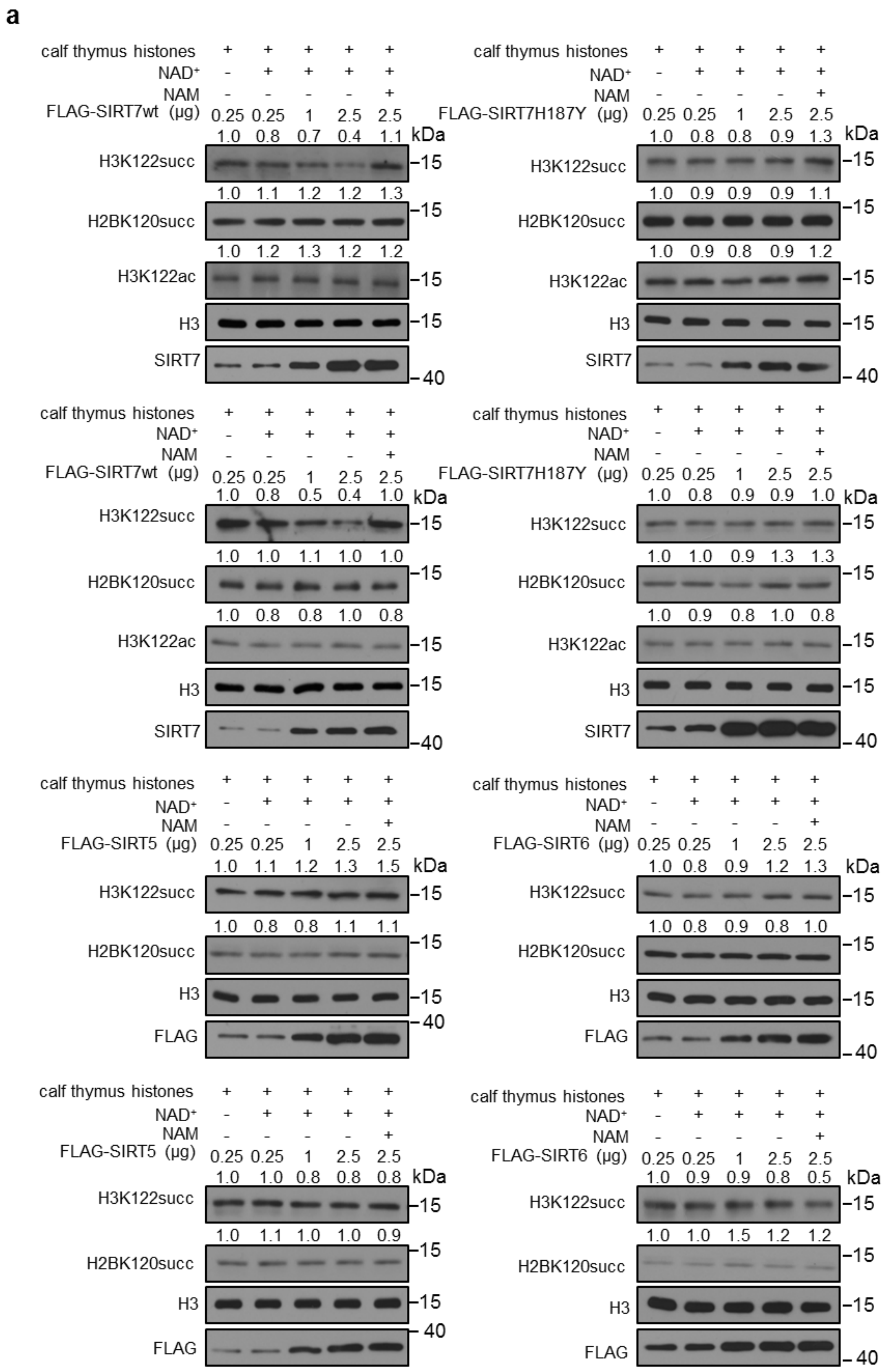

a
calf thymus histones + + + + +
NAD⁺ - + + + +
NAM - - - - +
FLAG-SIRT7wt (μg) 0.25 0.25 1 2.5 2.5
1.0 0.8 0.7 0.4 1.1
kDa
H3K122succ
1.0 1.1 1.2 1.2 1.3
H2BK120succ
1.0 1.2 1.3 1.2 1.2
H3K122ac
H3
SIRT7
15
40
calf thymus histones + + + + +
NAD⁺ - + + + +
NAM - - - - +
FLAG-SIRT7H187Y (μg) 0.25 0.25 1 2.5 2.5
1.0 0.8 0.8 0.9 1.3
H3K122succ
1.0 0.9 0.9 0.9 1.1
H2BK120succ
1.0 0.9 0.8 0.9 1.2
H3K122ac
H3
SIRT7
calf thymus histones + + + + +
NAD⁺ - + + + +
NAM - - - - +
FLAG-SIRT7wt (μg) 0.25 0.25 1 2.5 2.5
1.0 0.8 0.5 0.4 1.0
H3K122succ
1.0 1.0 1.1 1.0 1.0
H2BK120succ
1.0 0.8 0.8 1.0 0.8
H3K122ac
H3
SIRT7
calf thymus histones + + + + +
NAD⁺ - + + + +
NAM - - - - +
FLAG-SIRT7H187Y (μg) 0.25 0.25 1 2.5 2.5
1.0 0.8 0.9 0.9 1.0
H3K122succ
1.0 1.0 0.9 1.3 1.3
H2BK120succ
1.0 0.9 0.8 1.0 0.8
H3K122ac
H3
SIRT7
calf thymus histones + + + + +
NAD⁺ - + + + +
NAM - - - - +
FLAG-SIRT5 (μg) 0.25 0.25 1 2.5 2.5
1.0 1.1 1.2 1.3 1.5
H3K122succ
1.0 0.8 0.8 1.1 1.1
H2BK120succ
H3
FLAG
calf thymus histones + + + + +
NAD⁺ - + + + +
NAM - - - - +
FLAG-SIRT6 (μg) 0.25 0.25 1 2.5 2.5
1.0 0.8 0.9 1.2 1.3
H3K122succ
1.0 0.8 0.9 0.8 1.0
H2BK120succ
H3
FLAG
calf thymus histones + + + + +
NAD⁺ - + + + +
NAM - - - - +
FLAG-SIRT5 (μg) 0.25 0.25 1 2.5 2.5
1.0 1.0 0.8 0.8 0.8
H3K122succ
1.0 1.1 1.0 1.0 0.9
H2BK120succ
H3
FLAG
calf thymus histones + + + + +
NAD⁺ - + + + +
NAM - - - - +
FLAG-SIRT6 (μg) 0.25 0.25 1 2.5 2.5
1.0 0.9 0.9 0.8 0.5
H3K122succ
1.0 1.0 1.5 1.2 1.2
H2BK120succ
H3
FLAG

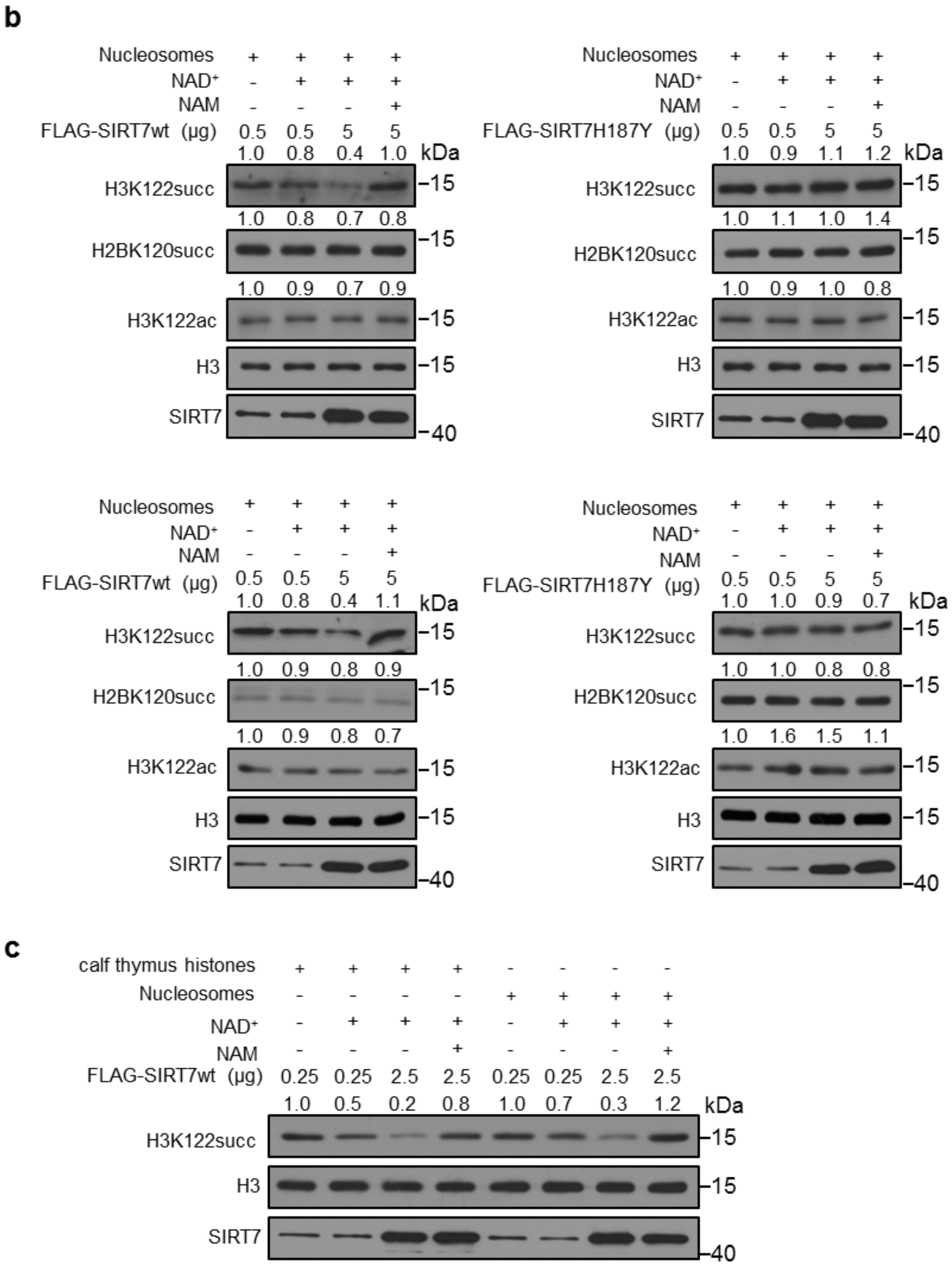


**Supplementary Figure 5. SIRT7 catalyzes histone H3K122 desuccinylation *in vitro.***

(**a**) *In vitro* desucinylation assays with calf thymus histones. Different amounts of purified FLAG-SIRT7wt, FLAG-SIRT7H187Y, FLAG-SIRT5wt, or FLAG-SIRT6wt were incubated with 1 μg calf thymus histones in the presence of 1.0 mM $NAD^{+}$ and/or 10 mM NAM. The reaction mixtures were boiled and analyzed by western blotting with the indicated antibodies. (**b**) *In vitro* desucinylation assays with mononucleosomes. Different amounts of purified FLAG-SIRT7wt or

FLAG-SIRT7H187Y were incubated with 1 μg HeLa cells-derived mononucleosomes in the presence or absence of 1.0 mM $NAD^+$ and/or 10 mM NAM. The reaction mixture was analyzed by western blotting with the indicated antibodies. (**c**) *In vitro* desucinylation assays with calf thymus histones and mononucleosomes. Different amounts of purified FLAG-SIRT7wt were incubated with 1 μg calf thymus histones or 1 μg HeLa cells-derived mononucleosomes in the presence or absence of 1.0 mM $NAD^+$ and/or 10 mM NAM. The reaction mixture was analyzed by western blotting with the indicated antibodies.

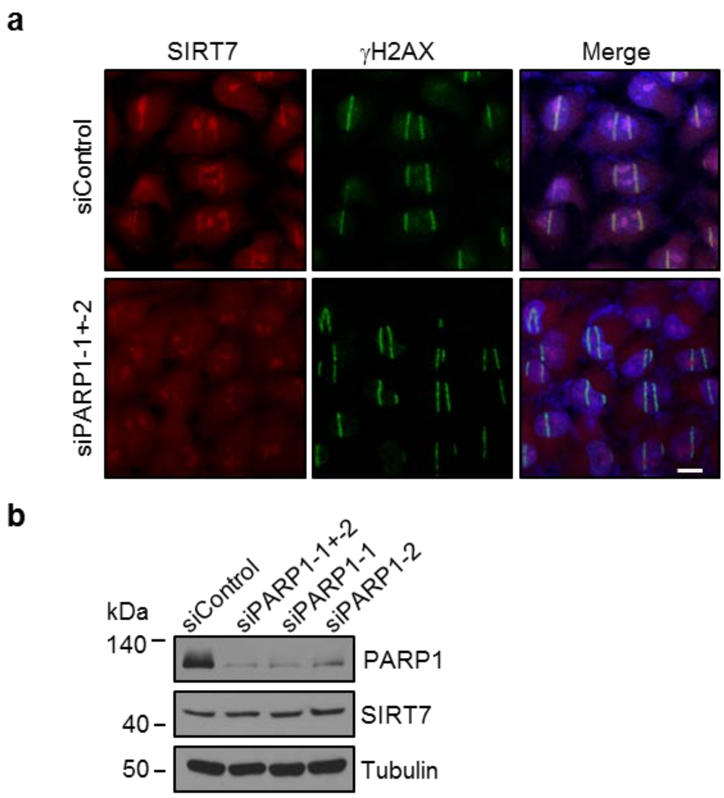


**Supplementary Figure 6. Recruitment of SIRT7 to DNA-damage sites is PARP1-dependent .**

(**a**) U2OS cells transfected with control siRNA or pooled siPARP1 (siPARP1-1 and siPARP1-2) were subjected to laser microirradiation and immunofluorescent analysis of SIRT7 and γH2AX at 5 min after microirradiation (upper). Bar, 10 µm. (**b**) Knockdown efficiency of PARP1 and the expression of SIRT7 were examined by western blotting.

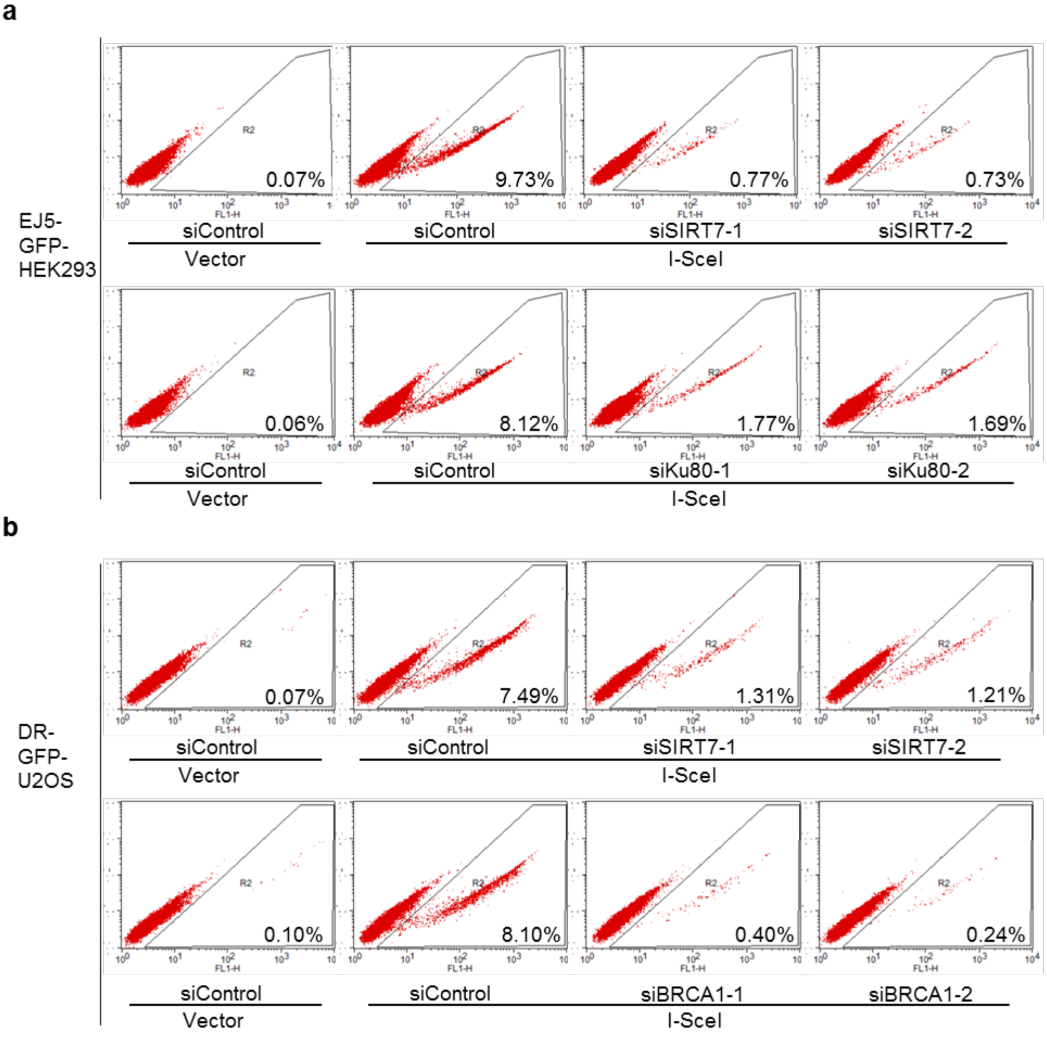


**Supplementary Figure 7. SIRT7 is required for efficient DSB repair.**

(**a**) NHEJ efficiency was determined by FACS in SIRT7- or Ku80-deficient EJ5-HEK293 cells. The partial original FACs plots in Fig. 5a were shown. (**b**) HR efficiency was determined by FACS in SIRT7- or BRCA1-deficient DR-GFP-U2OS cells. The partial original FACs plots in Fig. 5b were shown.

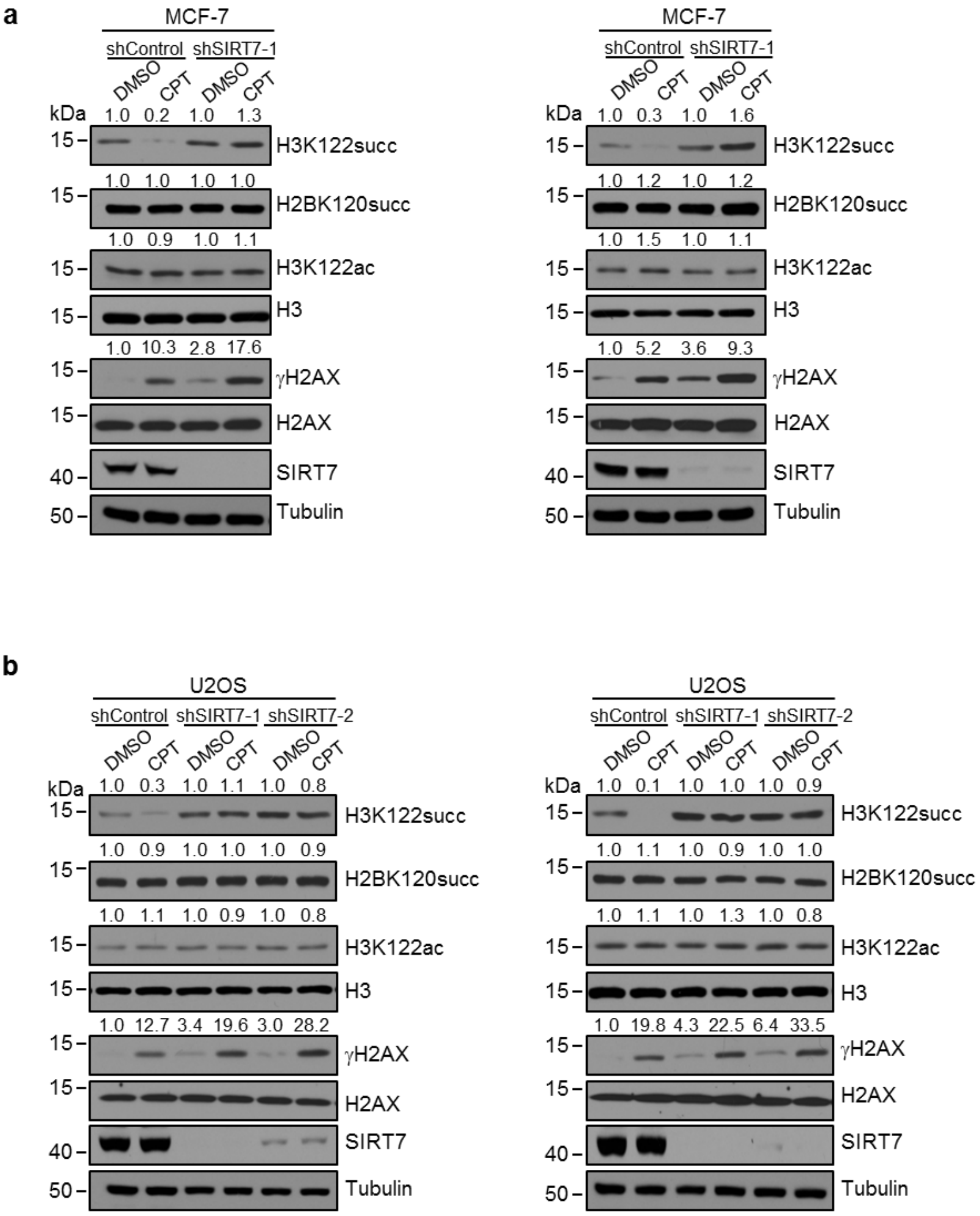

a
MCF-7
shControl
shSIRT7-1
DMSO
CPT
DMSO
CPT
kDa
1.0 0.2 1.0 1.3
H3K122succ
1.0 1.0 1.0 1.0
H2BK120succ
1.0 0.9 1.0 1.1
H3K122ac
H3
1.0 10.3 2.8 17.6
γH2AX
H2AX
SIRT7
Tubulin
15
40
50
MCF-7
shControl
shSIRT7-1
DMSO
CPT
DMSO
CPT
kDa
1.0 0.3 1.0 1.6
H3K122succ
1.0 1.2 1.0 1.2
H2BK120succ
1.0 1.5 1.0 1.1
H3K122ac
H3
1.0 5.2 3.6 9.3
γH2AX
H2AX
SIRT7
Tubulin
b
U2OS
shControl
shSIRT7-1
shSIRT7-2
DMSO
CPT
DMSO
CPT
DMSO
CPT
kDa
1.0 0.3 1.0 1.1 1.0 0.8
H3K122succ
1.0 0.9 1.0 1.0 1.0 0.9
H2BK120succ
1.0 1.1 1.0 0.9 1.0 0.8
H3K122ac
H3
1.0 12.7 3.4 19.6 3.0 28.2
γH2AX
H2AX
SIRT7
Tubulin
U2OS
shControl
shSIRT7-1
shSIRT7-2
DMSO
CPT
DMSO
CPT
DMSO
CPT
kDa
1.0 0.1 1.0 1.0 1.0 0.9
H3K122succ
1.0 1.1 1.0 0.9 1.0 1.0
H2BK120succ
1.0 1.1 1.0 1.3 1.0 0.8
H3K122ac
H3
1.0 19.8 4.3 22.5 6.4 33.5
γH2AX
H2AX
SIRT7
Tubulin

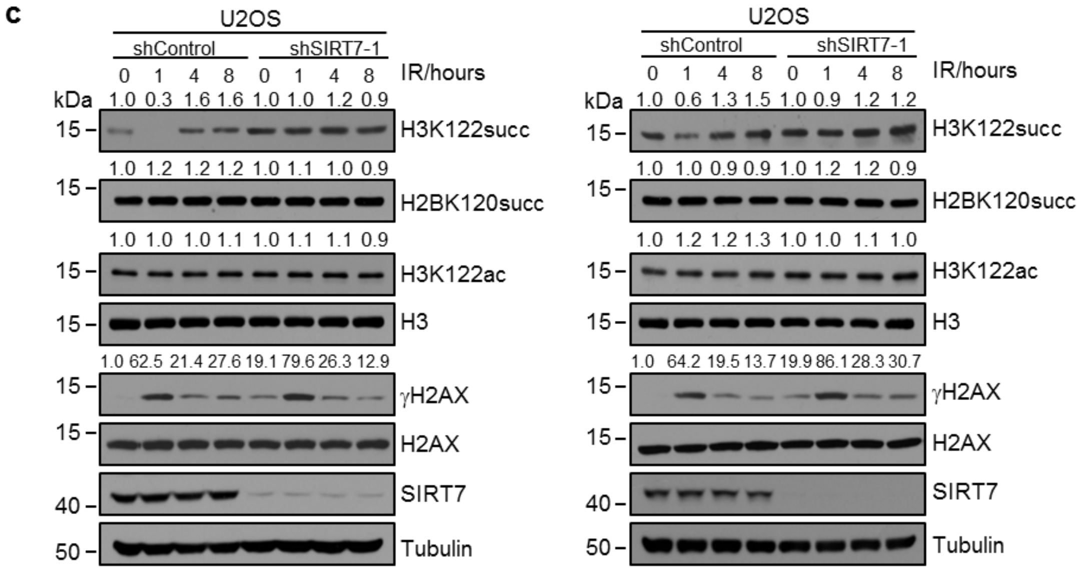


**Supplementary Figure 8. SIRT7 regulates H3K122succ in response to DNA damage reagents.**

(**a-b**) Control or SIRT7-depleted MCF-7 cells (**a**) or U2OS cells (**b**) were treated with 1 μM CPT for 8 h followed by histone extraction and succinylation and acetylation analysis by western blotting. The efficiency of SIRT7 knockdown and DNA damage effect induced by CPT were monitored by western blotting of whole cell lysate using antibodies against SIRT7 and γH2AX, respectively. (**c**) Control or SIRT7-depleted U2OS cells were exposed to 10 Gy of IR and collected at different time points for histone extraction and succinylation and acetylation analysis by western blotting. The efficiency of SIRT7 knockdown and IR treatment was monitored by western blotting of whole cell lysate using antibodies against SIRT7 and γH2AX, respectively.

a

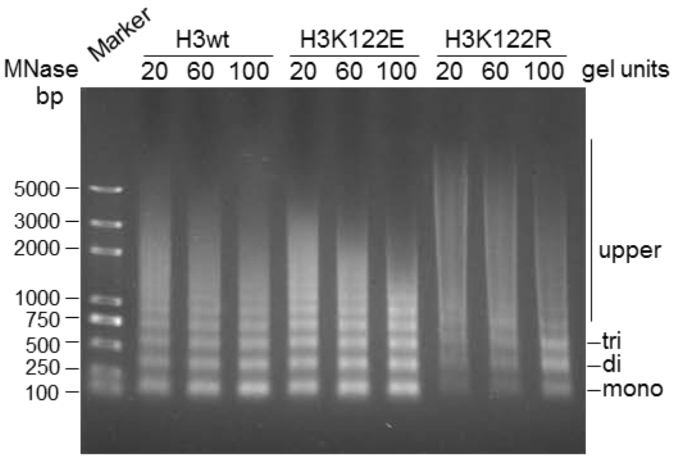
Marker
H3wt
H3K122E
H3K122R
MNase
20 60 100 20 60 100 20 60 100 gel units
bp
5000
3000
2000
1000
750
500
250
100
upper
tri
di
mono

b

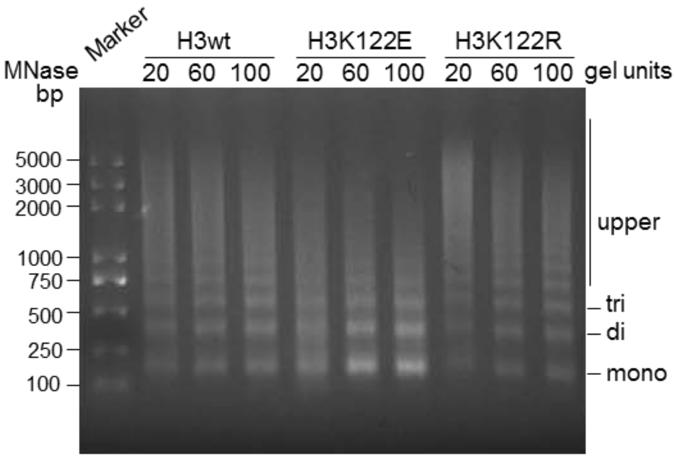
Marker
H3wt
H3K122E
H3K122R
MNase
20 60 100 20 60 100 20 60 100 gel units
bp
5000
3000
2000
1000
750
500
250
100
upper
tri
di
mono

c

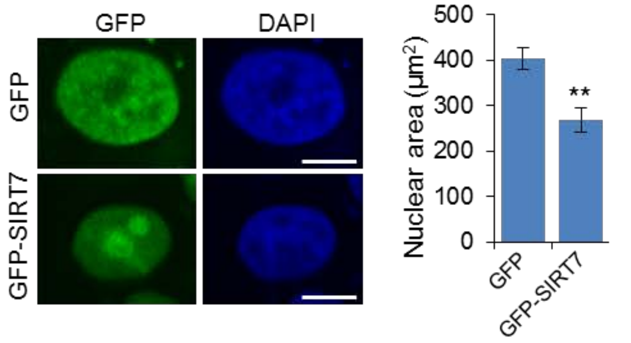
GFP
DAPI
GFP
GFP-SIRT7
Nuclear area (μm²)
500
400
300
200
100
0
**
GFP
GFP-SIRT7

d

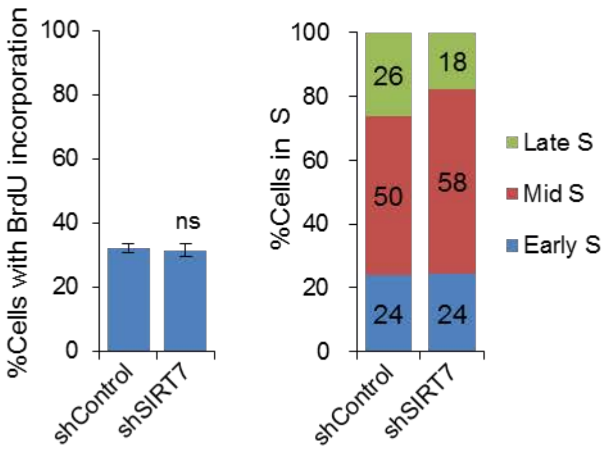
%Cells with BrdU incorporation
100
80
60
40
20
0
ns
shControl
shSIRT7
%Cells in S
26
18
50
58
24
24
Late S
Mid S
Early S

e

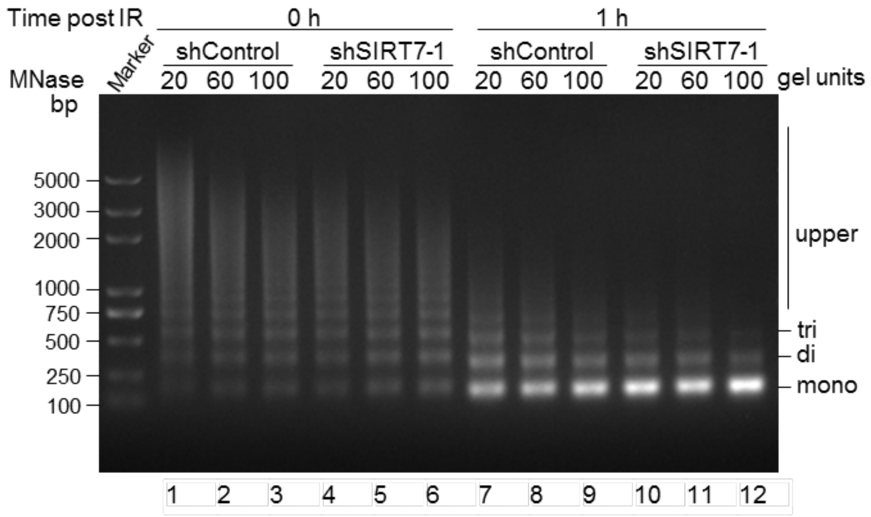


f

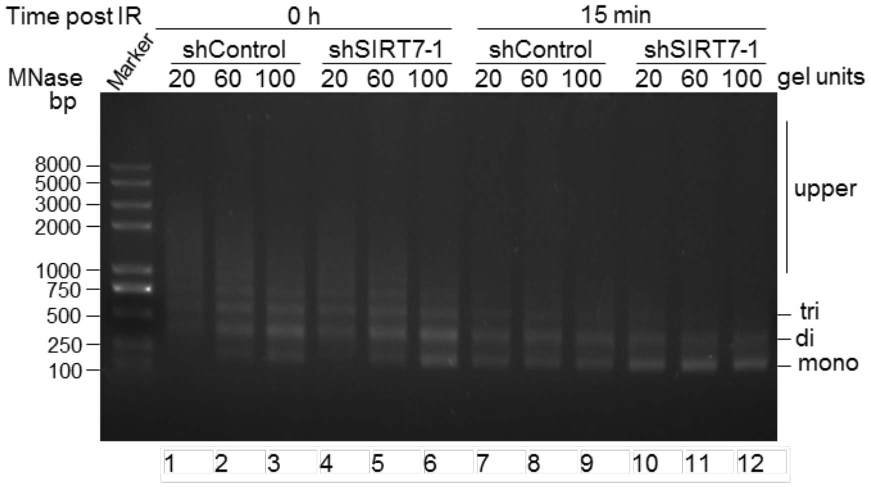


**Supplementary Figure 9. SIRT7-catalyzed H3K122succ desuccinylation is linked to chromatin condensation during DNA damage response.**

(**a-b**) Nuclei from U2OS cells stably expressing FLAG-H3wt, FLAG-H3K122E, or FLAG-H3K122R were incubated with 20, 60, or 100 gel units of MNase for 5 min followed by DNA extraction and ethidium bromide staining. The results of two replicates were shown. (**c**) U2OS cells expressing either GFP or GFP-SIRT7 (left). Scale bar: 10 μm. Quantification of the nuclear area is defined by DAPI staining (right). At least 30 independent cells were scored. Data are represented as mean ± SEM. (**d**) Quantification of positive cells for BrdU incorporation among control or SIRT7-depleted U2OS cells (left). Each bar represents the mean ± SD for triplicate experiments. BrdU positive cells were quantified in early, mid or late S phase according to their staining pattern in control or SIRT7-depleted U2OS cells (right). (**e-f**) Control or SIRT7-depleted U2OS cells were exposed to 10 Gy of IR and collected at 1 h (**e**) or 15 min (**f**). Nuclei were prepared and subjected to MNase assays with different amount of MNase. Mononucleosome, dinucleosome, and

trinucleosome are indicated. *$p < 0.05$ and **$p < 0.01$ (two-tailed unpaired Student's t test).

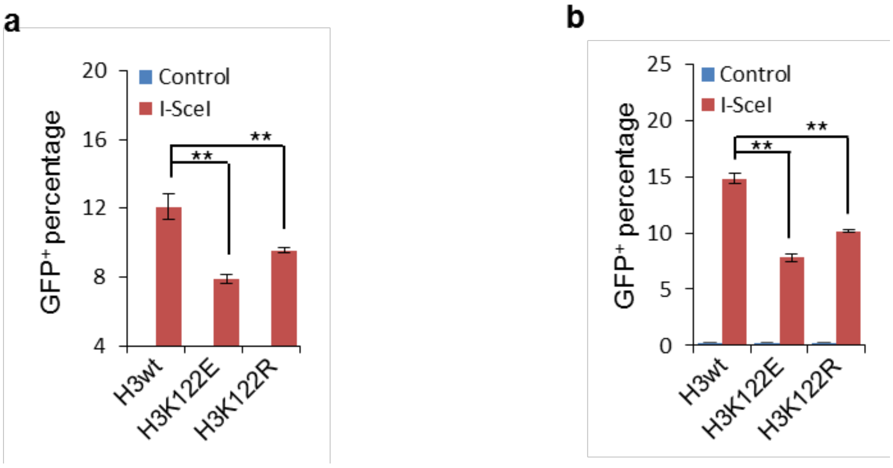


**Supplementary Figure 10. Overexpression of H3K122 mutants affected the repair efficiency of NHEJ.**

(**a-b**) Two independent repeats of cell flow cytometry experiments in EJ5-HEK293 cells. EJ5-GFP-HEK293 cells stably expressing FLAG-H3wt, FLAG-H3K122E, or FLAG-H3K122R were transfected with I-SceI for 48 h and analyzed by FACS. Each bar represents the mean ± SD for triplicate experiments. *$p < 0.05$ and **$p < 0.01$ (two-tailed unpaired Student’s t test).

Uncropped blots related to **Fig.1b**

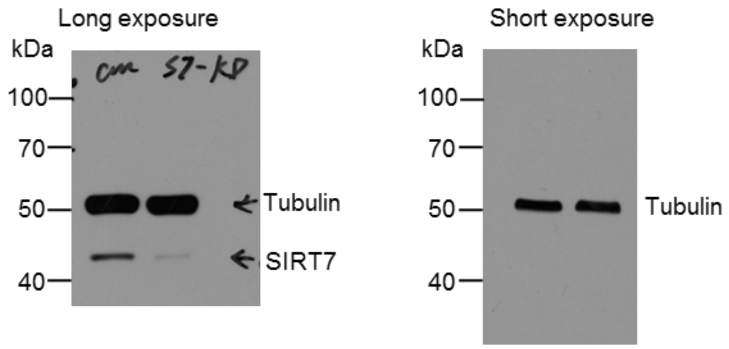


Uncropped blots related to **Fig.1d**

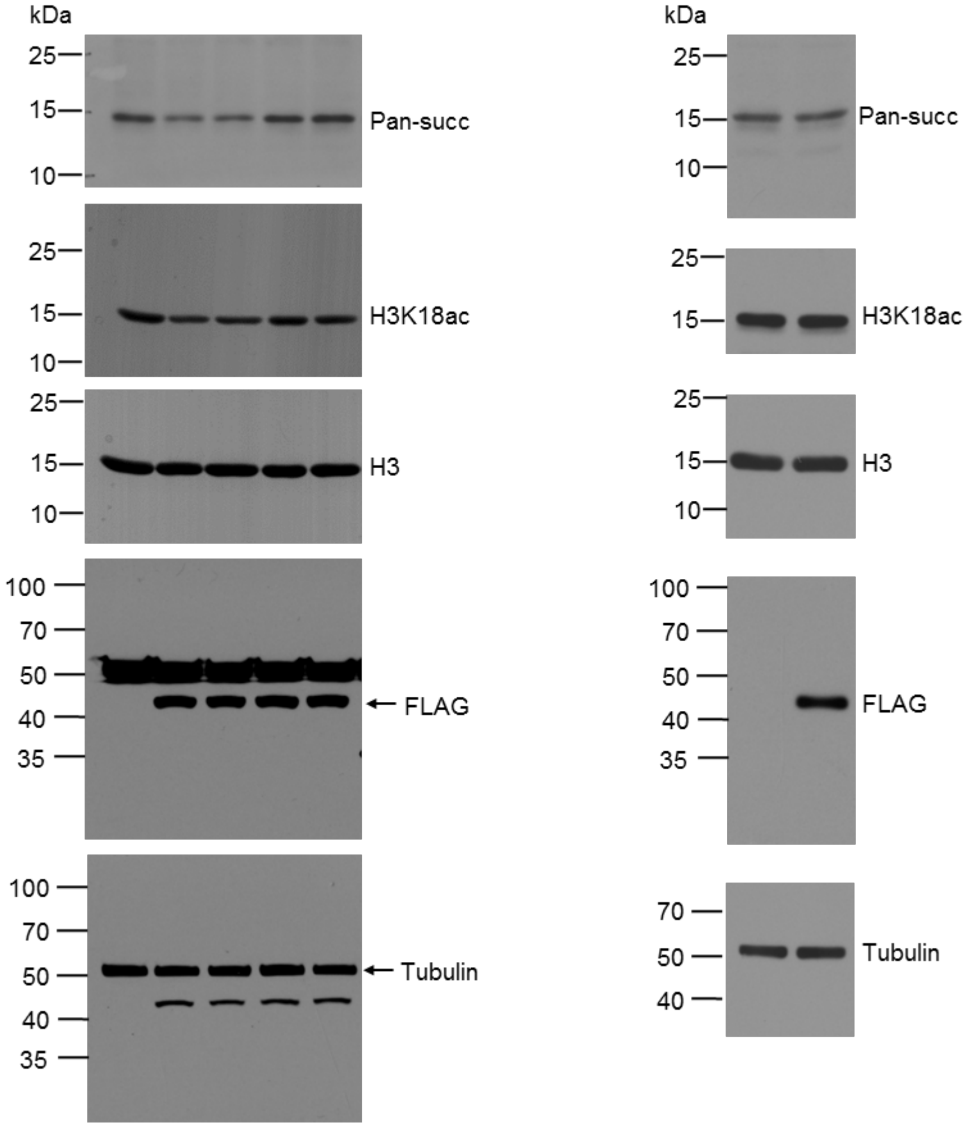

Uncropped blots related to **Fig. 2b**

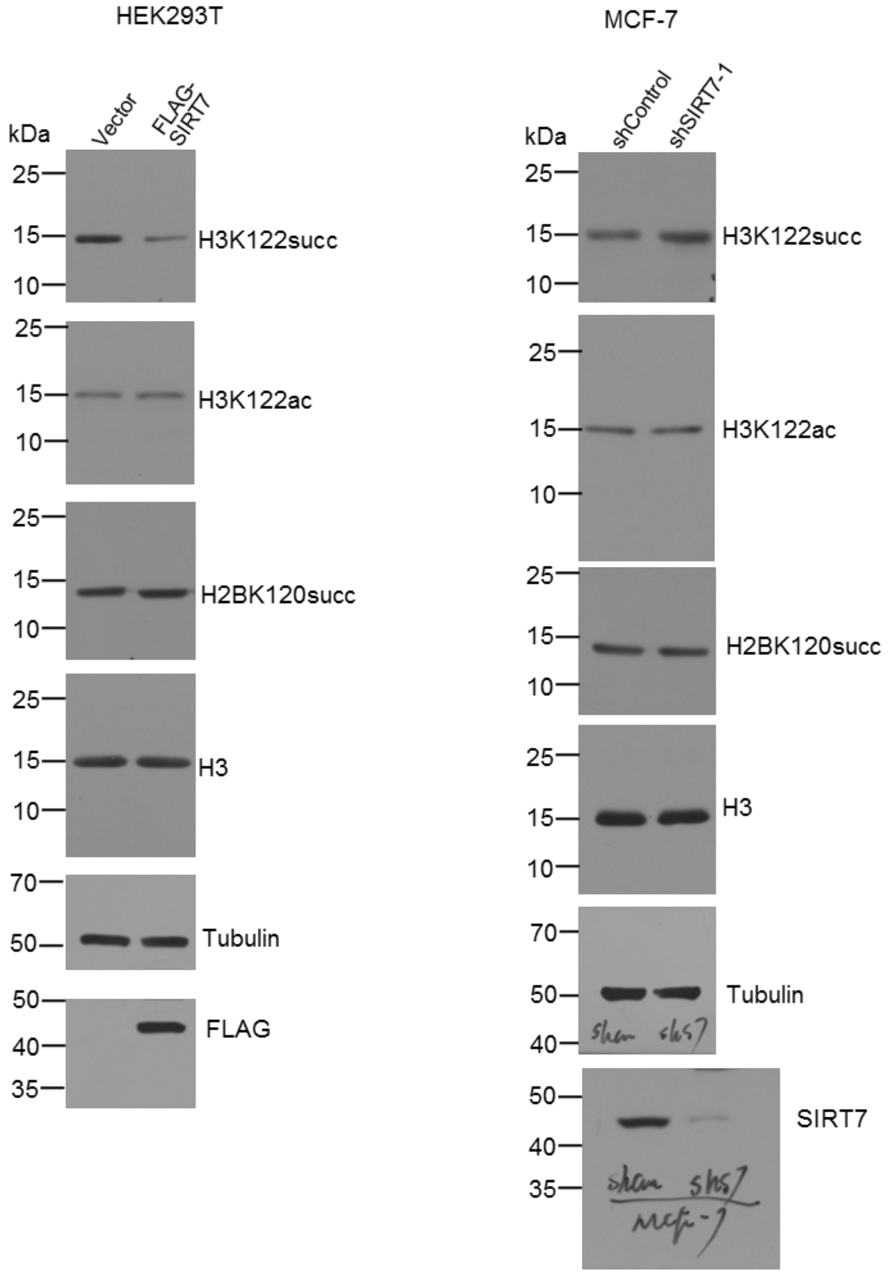

Uncropped blots related to **Fig. 2b**

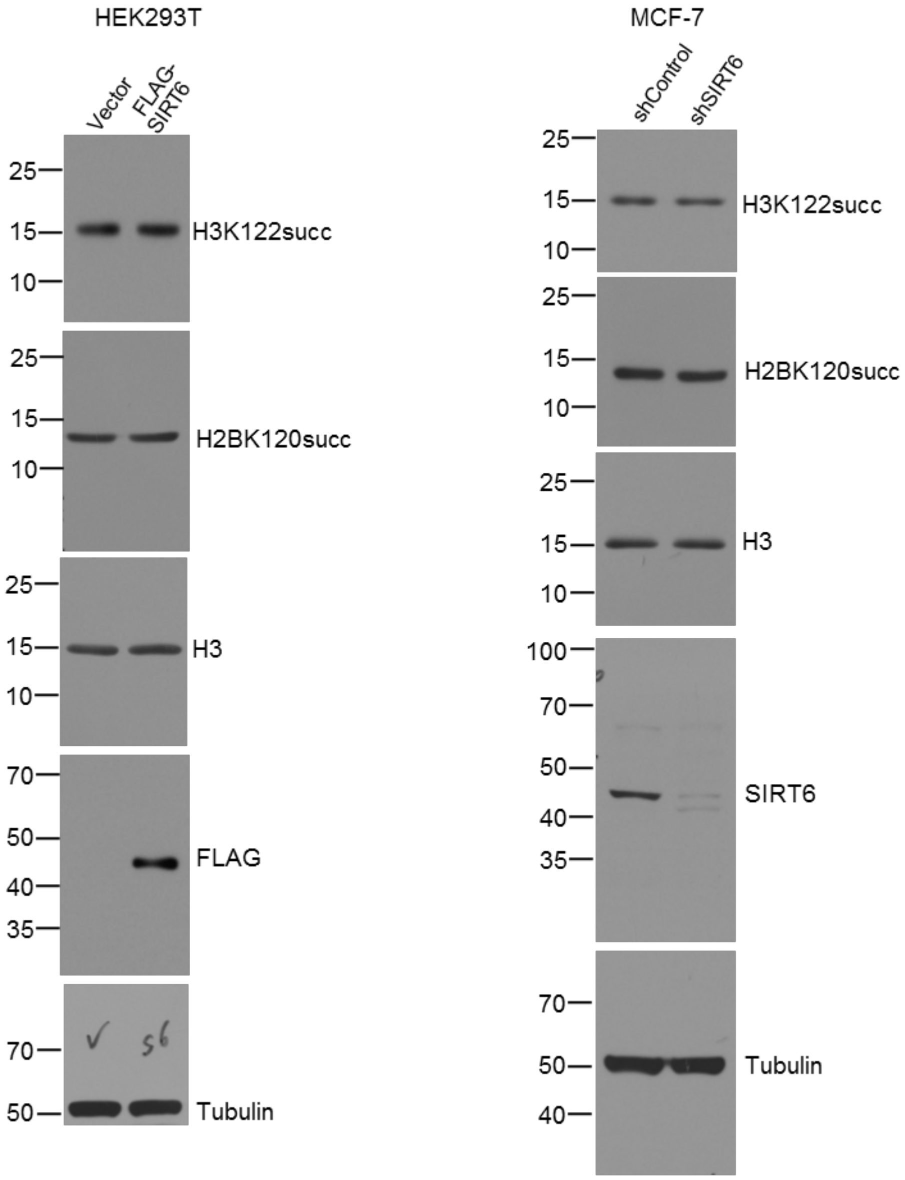


Uncropped blots related to **Fig. 2c**

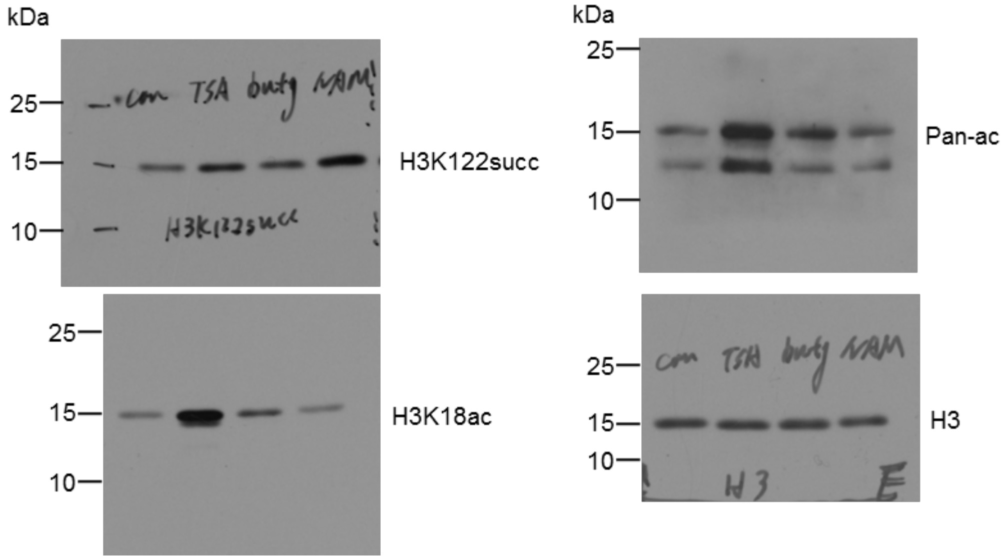

Uncropped blots related to **Fig. 3c**

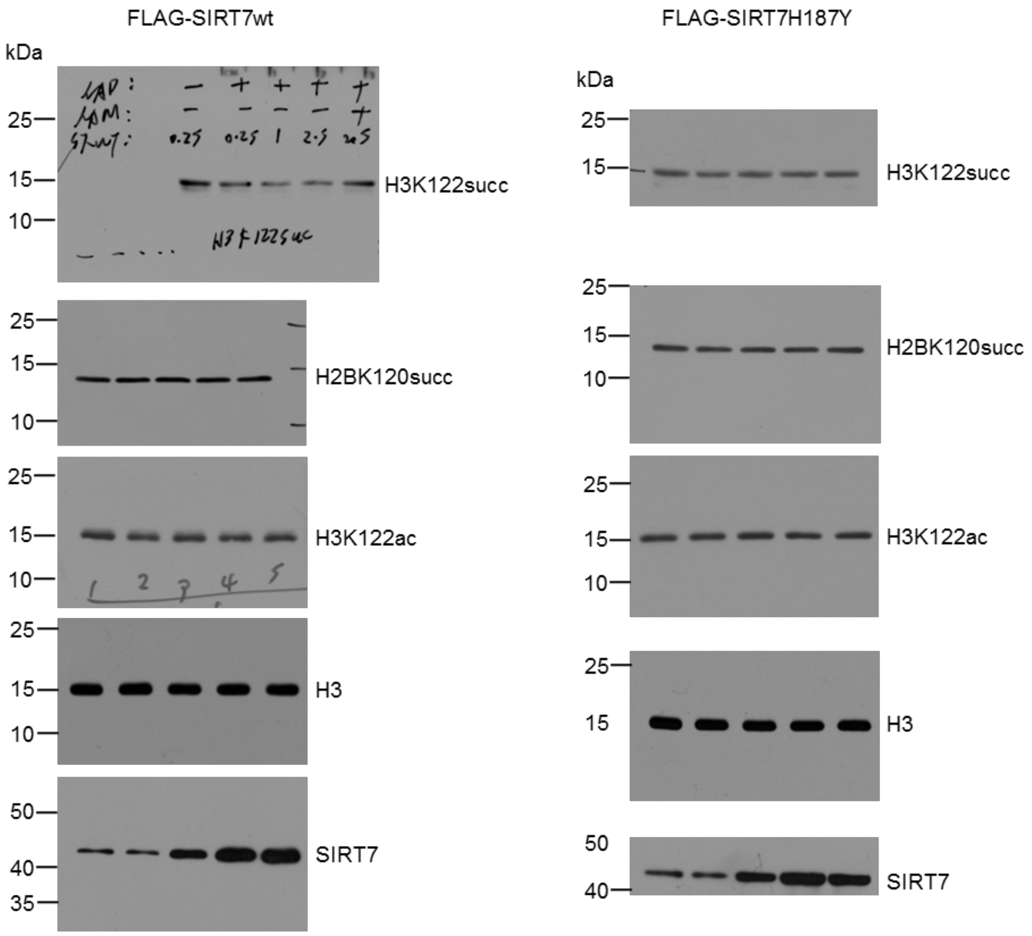

Uncropped blots related to **Fig. 3c**

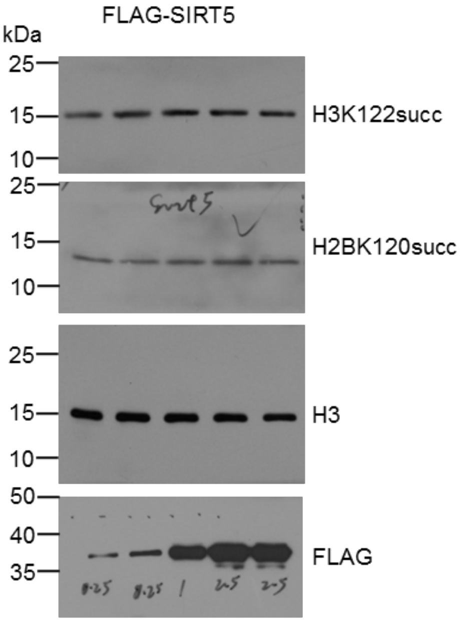


Uncropped blots related to **Fig. 3d**

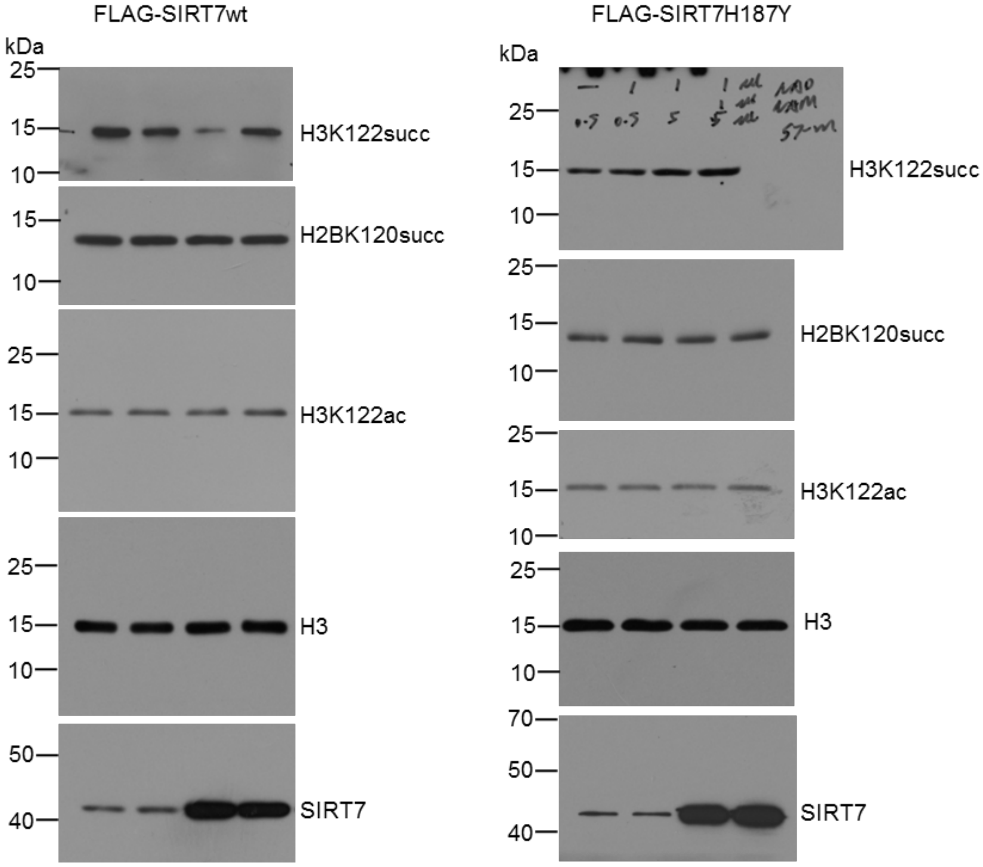

Uncropped blots related to **Fig. 4c**

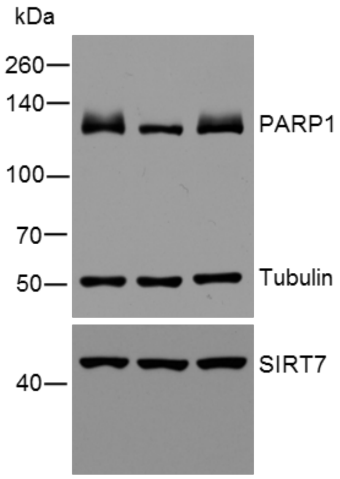


Uncropped blots related to **Fig. 6c**

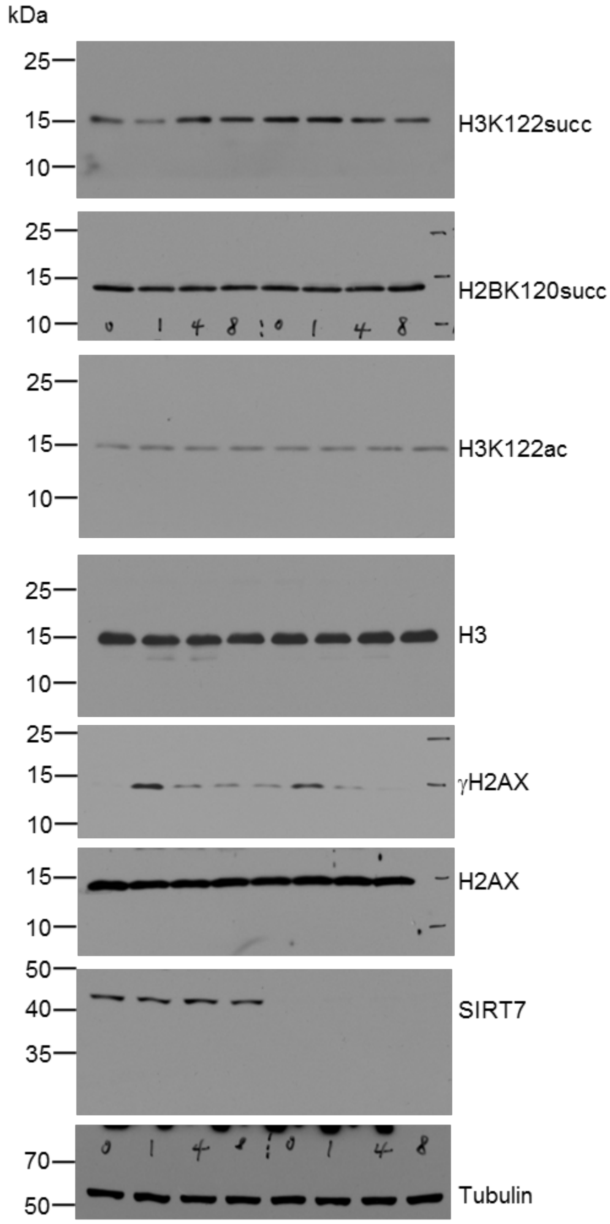

Uncropped blots related to **Fig. 7b**

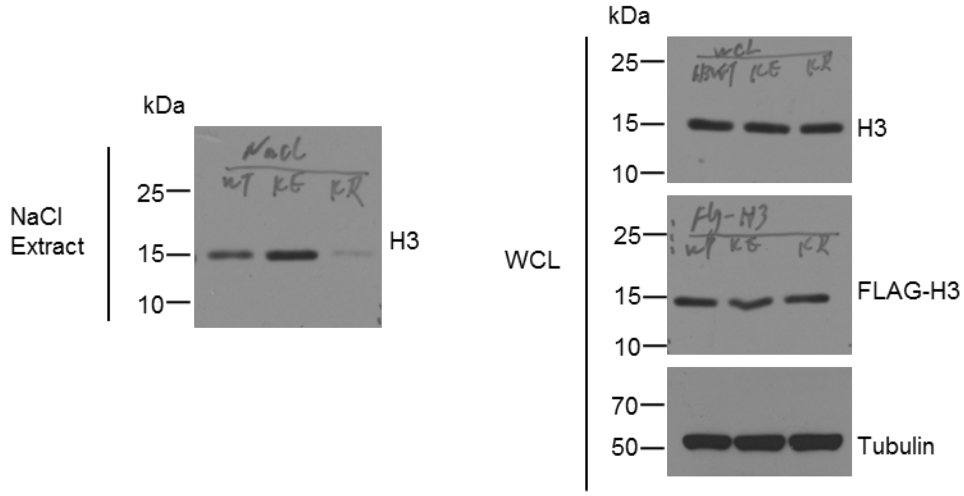


Uncropped blots related to **Fig. 7d**

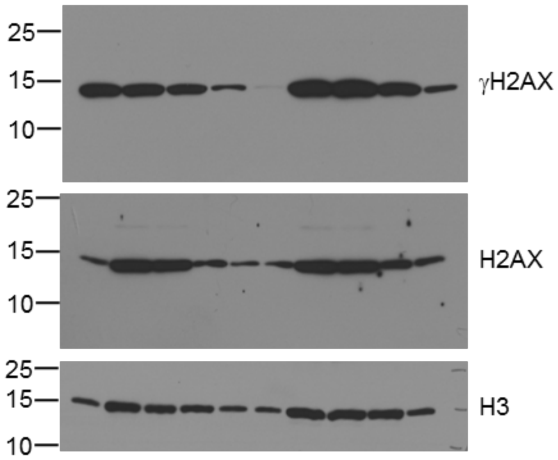


**Supplementary Fiigure 11. Unncropped blots related to Fig 1-7.**

**Supplementary Table 1. Chemically synthesized siRNA sequences**

| siRNA names | sequence |
|---|---|
| siSIRT7-1 | 5’-GGGAGUACGUGCGGGUGUUdTdT-3’ |
| siSIRT7-2 | 5’-CCCUGAAGCUACAUGGGAAdTdT-3’ |
| siKu80-1 | 5’-GCGAGUAACCAGCUCAUAAUU-3’ |
| siKu80-2 | 5’-AAGAGCUAAUCCUCAAGUCUU-3’ |
| siBRCA1-1 | 5’-GGAACCTGTCTCCACAAAG-3’ |
| siBRCA1-2 | 5’-GATAGTTCTACCAGTAAA-3’ |
| siPARP1-1 | 5’-GCATGATTGACCGCTGGTA-3’ |
| siPARP1-2 | 5’-GATAGAGCGTGAAGGCGAA-3’ |

**Supplementary Table 2. Lentiviral shRNA sequences**

| shRNA names | Strand | Sequence |
|---|---|---|
| ShControl | F | 5'-CCGGGAATCGTCGTATGCAGTGAAACTCGAGTTTCACTGCATACGACGATTCTTTTTG-3' |
| shControl | R | 5'-AATTCAAAAAGAATCGTCGTATGCAGTGAAACTCGAGTTTCACTGCATACGACGATTC-3' |
| shSIRT7-1 | F | 5'-CCGGAGAACGGAACTCGGGTTATTTCTCGAGAAATAACCCGAGTTCCGTTCTTTTTTG-3' |
| shSIRT7-1 | R | 5'-AATTCAAAAAAGAACGGAACTCGGGTTATTTCTCGAGAAATAACCCGAGTTCCGTTCT-3' |
| shSIRT7-2 | F | 5'-CCGGGTCCAGCCTGAAGGTTCTAAACTCGAGTTTAGAACCTTCAGGCTGGACTTTTTG-3' |
| shSIRT7-2 | R | 5'-AATTCAAAAAGTCCAGCCTGAAGGTTCTAAACTCGAGTTTAGAACCTTCAGGCTGGAC-3' |
| shSIRT6 | F | 5'-CCGGCTCCCTGGTCTCCAGCTTAAACTCGAGTTTAAGCTGGAGACCAGGGAGTTTTTG-3' |
| shSIRT6 | R | 5'-AATTCAAAAACTCCCTGGTCTCCAGCTTAAACTCGAGTTTAAGCTGGAGACCAGGGAG-3' |

**Supplementary Table 3. qChIP primers**

| Primer Names | Strand | Sequence |
|---|---|---|
| I-SceI | F | GATGGCACAGTGGTCAAGAGC |
| I-SceI | R | GAAGGATGGAAGGGTCAGGAG |

**Supplementary Table 4. sgRNA sequences**

| sgRNA names | sgRNA sequence |
|---|---|
| sgRNA-1 | CGTCCGGAACGCCAAATACT |
| sgRNA-2 | TGGAGATCCCCGCCTATAGC |
| sgRNA-3 | CAGCGTCTATCCCAGACTAC |
